# Evaluation of fatty acid‐related nutritional quality indices in processed and raw (*Lethrinus lentjan*) fish fillets

**DOI:** 10.1002/fsn3.3131

**Published:** 2022-11-09

**Authors:** Ali Aberoumand, Farideh Baesi

**Affiliations:** ^1^ Department of Fisheries, Natural Resources Faculty Behbahan Khatam Alanbia University of Technology Behbahan Iran

**Keywords:** fatty acid profile, *Lethrinus lentjan* fish, marinating, shelf life

## Abstract

The *Lethrinus lentjan* fish is economically and nutritionally important in Iran. Therefore, knowledge of their chemical composition can help in the development of functional foods. In the present study, proximate and fatty acid compositions were determined in fresh and marinated *Lethrinus lentjan* fish*.* Fatty acids were identified by gas chromatography. The 24 pieces of fresh *L. lentjan* fish with an average weight of 470 ± 125 g were prepared. After removing wastes, the fish fillet was placed in a dish containing acetic acid, salt, garlic, and red pepper. The marinade product contained n‐6PUFA (7.94%) and n‐3PUFA (3.46%). The results showed that moisture content in the marinade sample decreased, while fat, protein, and ash contents increased and carbohydrate content was also decreased compared to the raw sample. The marinating process increased PUFA percentage (19.32%) and ratio of PUFA and SFA (0.61). The fresh and processed samples showed superior nutritional quality and the lowest h/H ratio, but AI and TI ratios were relatively favorable (*p* < .05). The thrombogenicity index (TI) (0.46) and atherogenicity index (AI) (3.56) were more than the recommended in terms of risk of coronary heart disease. It can be concluded that fresh and processed fish samples represent an excellent source of high‐quality fat, demonstrating that this fish species’ freshwater can be considered optimal for human consumption.

## Graphical abstract

The marinade sample was rich in n‐6PUFA (7.94%) and n‐3PUFA (3.46%) with about one‐third of total fatty acids. The results showed that moisture content in the marinade sample decreased, while fat, protein, and ash contents increased and carbohydrate content was also decreased compared to the raw sample. The marinating process increased PUFA concentration (19.32%) and ratio of PUFA and SFA (0.61%). The fresh and processed samples showed superior nutritional quality and the lowest h/H ratio, but AI and TI ratios were relatively unfavorable (*p* < .05). The thrombogenicity index (TI) (45.28) and atherogenicity index (AI) (3.56) were more than the recommended in terms of risk of coronary heart disease. It can be concluded that fresh and processed fish samples represent an excellent source of high‐quality fat, demonstrating that this fish species’ freshwater can be considered optimal for human consumption.

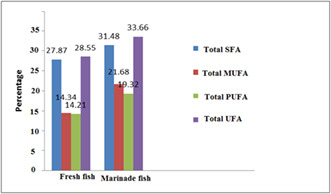

## 1 INTRODUCTION

The fish *Lethrinus lentjan* is one of the most popular sea fish in Iran, which has a delicious taste. People usually eat fried fish in oil, while they consume less processed fish products because there is no industrial marinating process for this type of fish in Iran. Annual fish consumption in Iran is low. The marinating process is a traditional preservation method that produces a product with acceptable taste, texture, and organoleptic properties. The nutritional and sensory quality of marinated fish is usually higher than that of fresh fish.

There is limited information on marinated seafood products. Fish preservation is not advanced with marinating technology. Therefore, the standardization method should be applied in the food industry with increasing useful life. The fish marinade is one of the fish products that consists of fresh, salted, or frozen fish or parts of fish that have been processed with a solution containing spices, sugar, plant extracts, oils, and acids. Vinegar and juice are used to improve tenderness, freshness, taste, and aroma to increase shelf life. The marinade process, which is a traditional method of preservation, is very popular due to their optimum texture and taste properties. Protecting fish with marinating technology is a traditional method in India. Therefore, the standard method should be applied in the food industry with increasing shelf life (Sushri et al., 2020).

Recently, seafood‐producing countries have focused on marinating the low‐consumption fish (Eysteinsson, 2016). The marinade is used for the fish. It is considered as a suitable semi‐preservative method for fish (Arason et al., 2014). Marinated fish products have a short shelf life and must be kept cold during processing and until consumption (Köse, 2010). Fish in different sizes and sections such as head, intestines, and fillets can be used in the process of marinating (Köse, 2010). Some fatty fish such as mackerel, herring, sardine, and anchovies are the main fishery products used (Arason et al., 2014). Arason et al. (2014) investigated on raw materials quality and compounds used in the marinating process. This means that the lipid content of the fish fillets, fishing methods, and handling methods may affect the quality of raw materials and marinated products. The poor quality of materials may directly affect the final products. Therefore, proper transportation, cooling, and fast postlanding processing lead to good quality, and also the suitable composition of materials used in the marinating solution can provide good quality marinated products.

Limitations such as gas spoilage, which appear with the protrusion of the sealed crucible, sludge formation and odor change during storage (Sushri et al., 2020), which are related to organisms such as acetic‐tolerant lactic acid bacteria such as *Leuconostoc gelidum* and *Leuconostoc gasicomitatium*. Therefore, before adding vegetables and spices, it should be sterilized or stored in the refrigerator to kill microorganisms.

Marinating is a treatment processed with edible acids, salt, sauce, and oil. This includes increasing the ionic strength and lowering the pH, which creates a favorable change in the taste and texture of the marinades. Since most marinades contain acidic substances, they should be stored in glass, ceramic, or stainless steel crucible. Food should be covered and refrigerated during the process (Ozogul & Balikci, 2013).

The amounts of marinade ingredients led to noticeable changes in the mass balance of protein, sodium chloride, moisture, and carbohydrates, as well as in protein yields, during marinade processing and drying (Fernandes et al., 2016).

The majority of the fatty acid percentages were statistically different between the two fish species. The most abundant fatty acids in the by‐products from two fish species were oleic (18: 1), palmitic (16:0), linoleic acid (18:2), and eicosenoic (20:1) acids. The total fatty acid content as well as the content of saturated, monounsaturated, and polyunsaturated fatty acids was significantly higher in the gilthead sea bream by‐products (Aikaterini et al., 2020). The aim of the present research was to investigate the effects of marinating on the fat, protein, moisture, ash contents, composition of fatty acids, and quality of fresh *Lethrinus lentjan* fish.

## 2 MATERIALS AND METHODS

### 2.1 Samples preparation

This research was conducted in 2018 at Khatam Alanbia University of Technology, Behbahan, Iran. Four fresh Shahri (*Lethrinus lentjan*), a sea fish species, with each weighed approximately 470 ± 125 g were caught from waters of southern Iran. Fresh fish were placed inside box in layers of crushed ice about 5 cm thick and were transported to the laboratory within 1 h. Its skin was shiny and muscle was firm. The fish were beheaded, gutted, filleted, and washed separately. The fillets were washed under tap water and placed in a clean dish to dry. The fish fillet yield was 45%. The 900‐g fish total fillets were divided into two groups for control and marinade treatments. The amount of used fillet for each analysis of fatty acids in the two groups was 100 g. The remaining fillets were used for analysis of proximate composition. Each analysis was done in triplicate. The first group was immersed in a marinade mixture for 2 h at 4°C as follows. The second group was considered as a control group in the same conditions without marinating process.

### 2.2 Preparation of marinating mixture

In a bowl, 500 ml of acetic acid, 250 g of freshly grated garlic, 60 g of table salt, 3 g of red pepper, and 100 g of distilled water were mixed well as a marinating mixture. The marinating process was performed at 4°C. All marinated samples were placed separately in a sealed plastic container and incubated in the refrigerator (4°C) for 14 days and then analyzed.

### 2.3 Method of marinade preparation

The marinating method was cold marinating, in which the first one is the most commonly used (Arason et al., 2014). Cold marinating consists of treating the fish in a marinating bath with relatively high acetic acid (vinegar) and salt content (Capaccioni et al., 2011). The salt should be used in sufficient amount to keep the fish flesh firm, while the concentration of the vinegar will determine the degree of preservation. In addition, the fish should be fully immersed in the marinating solution throughout the process, this being one of the most important marinating conditions (Arason et al., 2014).

The first fish was washed with distilled water, and then all wastes were removed with a knife. The fish fillet prepared and cut into pieces 2 × 3 cm and then placed in 5% salt solution for 20 min (Moini et al., 2005). As much as 12% weight of the fish fillet, vinegar and salt are added. The fish pieces along with vinegar, salt, red pepper, and garlic were placed inside the glass crucible. Then, the glass crucible was closed and pasteurized in hot water. The pasteurization temperature was at 100°C for 15 min. The glass crucible was stored at temperature below 15°C. After 2 weeks, the fish was marinated. The marinade sample after processing were packed in oxygen‐protected and impenetrable crucible and then transported to the laboratory to measure fatty acids profile and proximate composition. The raw sample (control) was used for the analysis of proximate and fatty acids composition. All samples were analyzed in triplicate.

### 2.4 Determination of proximate composition

For measuring the protein, the sample was dried in an oven at 103°C for 30 min. The dried sample was crushed by an electric grinder. The powdered sample was used to determine the fillet proximate composition in the laboratory.

#### 2.4.1 Determination of moisture content

To determine moisture content, the crucible was placed in an oven (Parmis Azma, Iran) at 103°C for 30 min to dry. The crucible is then transferred to a desiccator and weighed after cooling. The 10 g of the sample was placed in the crucible and placed it in the oven at 103°C for 10 h. The plant containing the sample is placed in the dryer for 30 minutes and then weighed (AOAC, 2005). The results of each treatment showed as follows:

The percentage of the moisture content in the sample was calculated by the following formulae: % of moisture = {(weight of original sample – weight of dried sample)/weight of original sample} × 100.

#### 2.4.2 Determination of ash content

The 10 g of sample, which previously had been dried and weighed in the oven, was placed in a Crucible, and then placed in an electric furnace (Parmis Azma, Iran) at 500°C for 12 h. Then, they were placed in a desiccator for 30 min to cool. Then, the crucible was placed in the desiccator and weighed (AOAC, 2005). The weight of remained ash was determined based on the following formula:

$$\text{Percentage of}\mathrm{ash}=\left(\mathrm{ash}\text{weight}/\text{initial sample weight}\right)\times 100.$$

#### 2.4.3 Determination of crude fat

The 5 g of the sample dried in the oven and then was weighed with a scale of 0.01 g. The dried sample was placed on filter paper and after weighing, it was placed in the extractor of Soxhlet apparatus (Parmis Azma, Iran). A volume of 100 ml of ether was put into the balloon, and then it was connected to the refrigerant, and the fat was extracted by the heater at a temperature of 60°C for 8 h. The distillation of the solution continued until the balloon was free of solvent (AOAC, 2005). Then, samples were dried in a hood. The sample fat content was calculated using the following equation:

Fat%=extractedfatweight/initial sample weight×100.

#### 2.4.4 Determination of crude protein

The protein content of the samples was determined using the Kjeldahl method. One gram of the sample was placed in a digestion balloon and 150 ml of concentrated sulfuric acid was added to each balloon along with the catalyst. After placing the balloon in the apparatus, the sample was boiled at a low temperature for about 30 min to foam and then the temperature was raised to digest the sample. The sample took about 4 h to digest. After digestion and cooling of the samples, distilled water was added to each balloon and placed in the titration section of the Kjeldahl device (Parmis Azma, Iran) and titrated with 12 ml of concentrated sulfuric acid 0.1 N normal sulfuric acid. Total nitrogen was determined by the Kjeldahl method and then multiplied by 6.25 (AOAC, 2005), the obtained number indicated crude protein content.

The percentage of protein was calculated according to the equation below.

$$\%\text{Nitrogen}\left(\%N\right)=\left(T–B\right)\times N\times 14.007\times 100/\text{Weight of sample}\left(\mathrm{mg}\right).$$

% Protein = N × F Where: T, Titration volume for sample (ml); B, Titration volume for blank (ml); N, Normality of acid to 4 decimal places; F, Conversion factor for nitrogen to protein (6.25).

#### 2.4.5 Determination of carbohydrate content

Carbohydrate content was determined based on difference calculation: [%carbohydrate =100 – (%moisture + %ash + %crude protein + %fat)].

#### 2.4.6 Determination of calorific value

The calorific value was determined indirectly using Rabner coefficients for aquatic organisms: 9 kcal/g for lipids, 4 kcal/g for proteins (Winberg, 1971), and in kcal wet‐weight by Eder and Lewis (2005) expressed.

### 2.5 Extraction of fat from fillets

A modification of the Folch method of lipid extraction was used (Washburn, 1989). The 1 g of the sample was transferred into a 50‐ml volumetric flask, and then 20‐ml chloroform was added to the sample and shaken vigorously. Then, 10 ml of chloroform was added to the sample and the Crucible was shaken vigorously again. The sample was placed at 4°C for 12–24 h completely so the fat was removed. After 24 h, the samples were transferred into the decanter and 5‐ml distilled water was added to it and transferred to the decanter. After one hour, separately three phases were formed inside the decanter, the fat and solvent phase, which was located in the lower part of the decanter, was transferred to the (COD) Chemical Oxygen Demand crucible by funnel and filter paper, and by liquid nitrogen, the solvent was separated and the fat remained.

### 2.6 Preparation of methyl ester fatty acid

First, homogenization was performed, and then a 0.1 g oil sample was weighed inside the test tube with a Pasteur pipette. Then, 2 ml of iso‐octane solvent was added, and then 100 μl from 2 molar 2 methanol was added to it. The test tube was closed and mixed vigorously for 1 min. The contents of the test tube remained stationary for 2 min. Then, 2‐ml saturated sodium chloride was added to it and the test tube was closed and mixed vigorously for 1 min. The upper phase (isooctane) was removed and put into the vial. One gram of sodium hydrogen sulfate was added to it and mixed. Finally, the filtered solution was put into a glass vial and 1 μl was injected into the gas chromatography device (Iranian National Standard, manufacturer) against a triglyceride (triundecanoin C11:0) internal standard. The oven temperature was maintained at 100°C for 1 min, programmed at 25°C/min to 100°C and after 1 min, it was raised to 240°C at a rate of 5°C/min and held for 2 min. Then, the temperature was raised to 250°C at a rate of 5°C/min and held for 10 min. Inlet and Flame Ionization Detector (FID) temperatures were set to 250°C and 270°C, respectively. Peak identification and response factor calculation were accomplished using a FAME standard mixture (Supelco 37 Component FAME mix, Sigma‐Aldrich, Darmstadt). By comparing the chromatogram retention time of unknown samples with chromatograms obtained from the standard solution of methyl‐esterified fatty acids, the fatty acid percentage in the fish tissue was identified and the results were reported as a percentage. Separation of FAMEs was accomplished on the Agilent 7890B model gas chromatograph device equipped with a CP‐Sill 88 capillary column (60 m, 0.25mmID, 0.20 Micro‐Meter df) (Agilent), H2 carrier, and 2 ml/min flow rate. The split ratio was set at 1/10, and the type of detector was (FID) flame ionization detector. Temperature sensors and injection site set at 270°C and 250°C, respectively, were used (Christie, 1973).

### 2.7 Fat nutritional quality indices

Data from fatty acid composition were used to calculate fat nutritional quality indices using three relevant indices: Atherogenicity index (AI) and Thrombogenicity index (TI) according to Ulbricht and Southgate (1991) with changes and Hypocholesterolemic/hypercholesterolemic ratio (h/H). The indices were calculated as follows:

$$\mathrm{AI}=\left(4\times C14:0+C16:0\right)/\left(\sum \text{MUFA}+\sum \left(n-6\right)+\sum \left(n-3\right)\right).$$

$$\mathrm{TI}=\left(C14:0+C16:0+C18:0\right)/\left[\left(0.5\times \text{ΣMUFA}\right)+\left(0.5\times \mathrm{\Sigma n}-6\text{PUFA}\right)+\left(3\times \mathrm{\Sigma n}-3\text{PUFA}\right)+\left(n-3/n-6\right)\right].$$

$$h/H=\left(C18:1n-9+C18:2n-6+C20:4n-6+C18:3n-3+C20:5n-3+C22:5n-3+C22:6n-3\right)/\left(C14:0+16:0\right).$$

### 2.8 Statistical analysis method

Data were analyzed using analysis of variance (ANOVA). The least significant difference procedure was applied to test for differences between means. Statistical analysis was carried out with SPSS 16.0.

## 3 RESULTS AND DISCUSSION

Table 1 shows that protein, fat, ash, and calorific values increased in the marinated *Lethrinus lentjan* fish except for moisture and carbohydrates. This was due to the effects of acid and salt and other additives on the fish because they are moisture absorbers. As moisture content was decreased, other nutrients were increased. The decrease in moisture content can be due to the presence of salt in the marinade sample, which may have replaced part of the water in the raw material (Adepoju et al., 2018). Protein in the marinade was significantly higher (*p* < .05) and was lowest in control samples. Bilgin et al. (2005) reported that the fresh gilthead sea bream (*Sparus aurata* L., 1758) fish had lower protein (16%), lipid (1.26%), and ash (0.59%) content and higher moisture content (78.72%). Turchini et al. (2007) showed Murray cod, *Maccullochella peelii* content of protein in the range from 16.6% to 19% and lipid content varying between 2.29 and 2.75% with a different dietary treatment. The moisture content decreased which was due to the loss of water and due to the penetration of salt into the fillet, which leads to an increase in the protein content (Colakoglu et al., 2011). Marinades have a significantly higher amount of crude protein (*p* < .05), because these treatments contained vinegar, which may help the process of “carination” faster, thus increasing the production of protein compounds in fish fillets. The results obtained in the present study were consistent with Sallam et al. (2007) who observed that the marinating process in Pacific saury (*Cololabis saira*) reduced moisture content and increased the other components compared to the raw samples. The protein values of the samples in this study were determined at 19.11 and 34.28% for control and marinade, respectively. This is due to the removal of moisture and water‐soluble proteins by the salt from the fillet during processing. This protein value in marinade sample was in dry matter.

**TABLE 1:** Proximate composition of fresh and processed fish (in percentage of the wet sample)

Proximate	Protein value	Fat value	Moisture value	Ash value	Carbohydrates value	Energy (kcal/100 g)
Control	19.11 ± 0.14^a^	3.42 ± 0.24^a^	74.73 ± 0.47^a^	2.19 ± 0.08^a^	0.55	142.66
marinated	34.28 ± 0.16^b^	4.14 ± 0.19^b^	57.90 ± 0.23^b^	3.52 ± 0.24^b^	0.16	222.36

*Note*: Nonsimilar letters in each column indicate a significant difference (*p* < .05).

There was a significant difference between the proximate composition of raw and marinated fish (*p* < .05). The difference between fatty acid composition in the marinated and fresh *Lethrinus lentjan* fish is shown in Table 2. The predominant fatty acids were C16:0, C18:0, C18:1, C18:2, C20:4, and C20:5. Palmitic acid (C16:0) was primary saturated fatty acid, whereas oleic acid (C18:1) was primary MUFAs. The major fatty acids identified as PUFA of the marinated *Lethrinus lentjan* were eicosapentaenoic acid (EPA, C20:5), linoleic acid (C18:2), and arachidonic acid (C20:4). In the present study, SFA percentage was higher than MUFA and PUFA percentage in the marinated *Lethrinus lentjan*. C18:3 n‐3 and C22:6 n‐3 FA are actually absent in the fish lipids.

**TABLE 2:** Fatty acid profile of raw and marinated *Lethrinus lentjan* (%)

Fatty acids	Raw *L. Lentjan*	Marinated *L. Lentjan*
C14:0	0.59 ± 0.01^b^	0.62 ± 0.02^a^
C15:0	0.38^b^	0.39^a^
C16:0	19.06 ± 0.36^a^	22.38 ± 0.01^b^
C17:0	1.11 ± 0.02^b^	1.19^a^
C18:0	6.60 ± 0.01^b^	6.80 ± 0.03^a^
C23:0	0.13^a^	0.10^b^
Total SFA	27.87	31.48
C16:1c	1.09 ± 0.02^a^	1.01^b^
C17:1c	0.42 ± 0.01^b^	0.46^a^
C18:1c (n‐9)	12.83 ± 0.05^b^	20.21 ± 0.04^a^
Total MUFA	14.34	21.68
C18:2c	1.18^b^	7.59 ± 0.05^a^
C18:3 n6	0.51 ± 0.57	0.11^b^
C20:2c	0.19^b^	0.22^a^
C20:3 n6	0.19^b^	0.20^a^
C20:3 n3	0.20 ± 0.20^a^	0.19^a^
C20:4 n6	8.06 ± 0.03^a^	7.58 ± 0.01^b^
C22:2 n6	0.14^b^	0.16^a^
(EPA) C20:5 n3	3.74 ± 0.01^a^	3.27^b^
Total PUFA	14.21	19.32
∑ n3	3.94	3.46
∑ UFA	28.55	33.66
∑ n6	8.90	8.05
PUFA/SFA	0.50	0.61
∑ n3/∑ n6	0.44	0.42
∑ n6/∑ n3	2.26	2.33

*Note*: Similar letters in each row indicate the absence of significant differences (*p* < .05). SFA, MUFA, n‐3 PUFA, and n‐6 PUFA are saturated fatty acids, monounsaturated fatty acids, polyunsaturated fatty acids from the n‐3 family, and polyunsaturated fatty acids from the n‐6 family, respectively.

### 3.1 The ratio between unsaturated fatty acids of PUFA to saturated fatty acids

Table 2 shows that the fatty acid ratio of PUFA to SFA in the marinated sample increased compared to the fresh fish. Its amount in the fresh sample was 0.50%, which increased to 0.61% in the marinated sample.

It can be said that amount of soluble salt increased the marinade fillet fat content (Yunus et al., 2013). The salt in the marinated product leads to the activation of iron ions in fat oxidation. The change in dry matter composition of the marinated product can be influenced by changes in the amount of moisture, which can be due to the exchange of marinade solution and water available in fish tissue (Hedayati Fard et al., 2016).

### 3.2 Total polyunsaturated fatty acids (PUFA) and Total monounsaturated fatty acid (MUFA)

In the present study, total saturated fatty acids in the marinade treatment were highest, but MUFA and PUFA percentages were lower than SFA, respectively. In the present study, percentages of saturated fatty acids in the marinated treatment showed a significant increase after 2 weeks of storage and its amount increased from 27.87% in the control sample to 31.48% in the marinated sample (*p* < .05).

Among the fatty acids in *Lethrinus lentjan* fish in the control and the marinated treatments, palmitic acid with 19.06% in the control sample and 22.38% in the marinated sample were the highest percentage. Other researchers showed that this fatty acid was the highest among aquatic saturated fatty acids (Ghosh & Mukhopadhyay, 2007), which is in agreement with results of the present study.

PUFA and MUFA fatty acids showed a significant increase (*p* < .05) during the marinating process. In a study conducted by Hedayati Fard et al. (2016), there was no significant difference between the profile of saturated and unsaturated fatty acids of silver marinade carp after 30 days of storage at 4°C. Although the amount of saturated fatty acids in the marinade sample increased compared to the fresh sample, while amounts of unsaturated fatty acids of MUFA and PUFA decreased, but there was no significant difference (*p* < .05), which was not in agreement with present study results, because in present study percentages of unsaturated fatty acids of MUFA and PUFA increased during the marinating process.

In a study by Hedayati Fard et al. (2016), the amount and ratio of n‐3 and n‐6 fatty acids in the marinade sample were lower than in the control sample, which is in agreement with the results of present study. The ratio between unsaturated fatty acids to saturated fatty acids increased in the present study. In the present study, the processing technique significantly increased the percentage of saturated fatty acids and percentage of MUFA and PUFA (*p* < .05). In addition, n‐3 to n‐6 ratio in the marinade sample was lower, also total percentage of n‐3 and n‐6 decreased. As a result of marinating, the SFA percentage in fish increased and its amount increased from 27.87% in the control sample to 31.48% in the marinated sample (*p* < .05).

Ozden (2005) reported that the fresh and the marinade fish species of anchovies and rainbow trout were found to contain the most abundant saturated fatty acids (C16:0), and highest of monounsaturated fatty acids belong C18:1 n‐9 fatty acids, which was in agreement with the present study. Rosa and Nunes (2004) stated that fatty acids C16:0, C18:1 n‐9, C20:5 n‐3, and C22:6 n‐3 were main fatty acids in fish tissue that were responsible for the flavor, special taste, and smell of the marinated products. A high ratio of n‐3 to n‐6 is often cited as an indicator of better nutritional value (Zhao et al., 2010). The ratio of ∑n‐3/∑n‐6 fatty acids is commonly used as an index for assessing the nutritional quality of fisheries products (Chen & Zhang, 2007; Kuley et al., 2008). Results obtained in the present study showed that percentage of fatty acids of 18:1, 18:2, and 16:0 in the produced product increased compared with a raw sample that was in agreement with the results of Aikaterini et al. (2020), while in the present study 20:2 and 20:3 percentage in marinade sample also increased.

The monoenoic acids of the muscle tissues of *Lethrinus lentjan* were dominated by CI8 chains. Three 18:1 isomers make up more than 60% of the monoenes. A wide variety of normal and branched monoenoic acids ranging in chain lengths from CLs to C23 were detected in trace amounts. The saturated fatty acids of the *Lethrinus lentjan* constitute about one‐third of the total fatty acids. The dominant member of this group is palmitic acid, which is the end product of the basic biosynthesis of fatty acids. Some stearic acid is present along with much smaller amounts of other saturated acids, both normal and branched, ranging in chain lengths from CI4 to C23 (Rawdah Tarik & Zamil EI‐Faer, 1994). These researchers’ results agreed with our work results, meanwhile, the species were the same.

The PUFA/SFA, n‐3/n‐6, and n‐6/n‐3 ratios are used as useful indices for nutritional quality (Memon et al., 2011; Mert et al., 2015). According to Memon et al. (2011), an n‐3/n‐6 ratio considered beneficial for the human diet should be as higher as possible, and this ratio for control and the marinade samples in the present study was 0.44 and 0.45, respectively. Nutritionists recommend an n‐3 to n‐6 ratio of 1: 4 (Valencia et al., 2006). In the present study, this ratio in both the control and the marinated samples was in the range of recommended minimum (0.45). However, its amount in the marinade sample was very low difference compared to the control treatment. The results of a study conducted by Aberoumand and Baesi (2021) showed that the fish marinating process changed the nutritional value of the fish fillets.

The AI used for evaluating the fish species may be the main factor influencing the AI value, which ranges from 0.03 to 3.58., while AI values for the fish samples in the present study were 2.98 in control and 3.56 in treatment which were found in permissible levels. TI was used in many studies of fatty acid compositions to assess the degree of thrombogenicity. The TI was used due to the correlation between fatty acids and human health. The ranges of TI values for the fish samples in the present study were 0.46 in control and 0.40 in treatment. The Hh: Hypocholesterolemic/hypercholesterolemic ratio was used as one of the indices to evaluate product nutritional and health‐promoting aspects (Rincón‐Cervera et al., 2020). For fish, the value ranges from 1.54 to 4.83, while Hh values for fish samples in the present study were 0.60 in the control and 0.47 in the treatment which were found in permissible levels so it was lower than 4.83.

### 3.3 Nutritional quality index (NQI) of fresh and marinated fish fillets

The percentage of fatty acids in marinated and raw fish fillets is shown in Table 2. Fatty acid‐related nutritional quality indices can be n‐6/n‐3 ratio, PUFA/SFA ratio, Atherogenicity index (AI), Thrombogenicity index (TI), and Hypocholesterolemia/hypercholesterolemia index (Hh). The percentage of eicosapentaenoic acid (EPA) was 3.74% and 3.27% for raw and marinated fish, respectively. The ratio of unsaturated fatty acid and saturated fatty acid in raw fish was 0.50, which decreased to 0.61 in marinated fish. The ratio of n‐3 and n‐6 fatty acids was 0.44 in fresh fish and 0.42 in marinated fish. In the present study, the n‐6/n‐3 ratio was 2.33 for marinated fish and 2.26 for raw fish. Hence, this ratio increased significantly due to the marinating process. In the present study, AI and TI indices in marinated fish were 3.56 and 0.40, respectively, and in raw fish were 2.97 and 0.46, respectively. H/h index was 0.47 in marinated fish and 0.60 in raw fish. H/h ratio and TI indices in fresh fish are relatively lower in marinated fish (Table 3).

**TABLE 3:** Comparison of AI, IT, and h/H nutritional indices for raw *Lethrinus Lentjan* and marinated *L. Lentjan.*

Indices	Raw *L. Lentjan*	Marinated *L*.
AI	2.97	3.56
TI	0.46	0.40
h/H	0.60	0.47

The n‐6/n‐3 lower ratio indicates that food is very useful in preventing coronary heart disease, while a higher ratio increases this risk (Beigi et al., 2014). This ratio was 2.33 for marinated fish and 2.26 for raw fish in the present study. PUFA/SFA index is very useful for reflecting both effects of unsaturated fatty acids, mainly polyunsaturated and saturated. However, a ratio <0.45 is considered undesirable because higher may increase blood cholesterol (Estuary et al., 2020; Flores et al., 2018; Jayasena et al., 2018). In the present study, this ratio was 0.61 in marinated fish and 0.50 in raw fish. Therefore, this ratio was slightly lower than the desired ratio in the marinated fish.

Hence, lower values of AI, TI, and Hh indices are desirable, because they are considered to prevent cardiovascular diseases (Estuary et al., 2020; Karimian‐khosroshahi et al., 2016). In the present study, AI was higher in marinated fish fillets, indicating a lower nutritional quality of marinated fish compared to raw fish. Higher values of Hh index are considered desirable. Therefore, in the present study, this ratio was similar in the marinated sample (0.47) and raw sample (0.60) (Estuary et al., 2020; Karimian‐khosroshahi et al., 2016).

The percentage of polyenes in the muscle of fresh *Lethrinus lentjan* was 19.32%, somewhat more than in fresh fish (14.21%). There was a significant difference in the percentage of saturated fatty acids between the fresh (27.87%) and marinated (31.48%) fish. Monoenes in marinated fish were present at 14.34%, while in fresh fish, muscle was present at 14.34%. In both fresh and marinated fish, the predominant saturated fatty acid (SFA) was palmitic acid (16:0), the most abundant monounsaturated fatty acid (MUFA) was 18:1c and the predominant polyunsaturated fatty acids (PUFA) were C20:4 n6 and C20:5 n3. Thus, both fresh and marinated fish contained a high percentage of n‐3 long‐chain polyunsaturated fatty acids such as eicosapentaenoic acid (20:5 n‐3) and hence may contribute to the prevention of diseases related to geriatric and cardiovascular disorders and certain forms of cancer, among others (Heu et al., 2003). Rosa and Nunes (2004) reported that 16:0, 18:1 n‐9, 20:5 n‐3, and 22:6 n‐3 were the main fatty acids in aquatic organisms that are responsible for the peculiar taste and odor of marinated products.

The changes in predominant fatty acids that these authors have reported are similar to those found in the present study. Oxidation of a product during storage affects its quality due to the increase in the total percentage of saturated fatty acids and the decrease in the total percentage of unsaturated fatty acids (Agnieszka et al., 2021).

It is clear that marinated fish may not be sold after 24 h at room temperature, but frozen marinade fish can have a longer shelf life. The most observed finding was that rainbow trout could be frozen marinated for at least 56 days without any major unfavorable changes, and at the end of storage, both marinated and control samples could be consumed (Maktabi1 et al., 2015).

## 4 CONCLUSIONS

The analysis of the present study showed that the predominant saturated fatty acids were palmitic and stearic, while EPA, oleic acid, and arachidonic acid were the main unsaturated fatty acids. In contrast, unsaturated fatty acids were higher in marinated fish fillets. The ratio of n‐3/n‐6, Atherogenicity, and Thrombogenicity indices in marinated fish fillets was in favorable amounts of food quality. The Hypocholesterolemic/hypercholesterolemic ratio was relatively similar in fresh and marinated fish fillets. The present study results showed that marinating process increased nutritional and calorific values in the marinated fish. Proximate composition in the marinated *Lethrinus lentjan* significantly increased (*p* < .05), but moisture and carbohydrate contents decreased. The marinated fish fatty acids profile changed. The marinating process increased percentages of SFA and PUFA fatty acids and also the ratio between PUFA and SFA. Increasing SFA and PUFA percentages indicated that oxidation was low in progress, and this has a favorable effect on quality. In marinated fish products that contain high or medium fat, the oxidation due to enzymatic reactions causes a loss of quality during storage, even if preventive measures are taken before and during the marinating process.

## FUNDING INFORMATION

This research work was funded by Behbahan Khatam Alanbia University of Technology, Behbahan, Iran.

## CONFLICT OF INTEREST

The authors do not have any conflict of interest.

## ETHICAL APPROVAL

This study did not involve human or animal testing.

## INFORMED CONSENT

Written informed consent was obtained from all study participants.

## Data Availability

The datasets generated and analyzed during the current study are available from the corresponding author upon reasonable request.
